# Differences in Recruitment and Life-History Strategy Alter Zooplankton Spring Dynamics Under Climate-Change Conditions

**DOI:** 10.1371/journal.pone.0044614

**Published:** 2012-09-06

**Authors:** Mattias K. Ekvall, Lars-Anders Hansson

**Affiliations:** Aquatic Ecology Unit, Department of Biology, Lund University, Lund, Sweden; Federal University of Rio de Janeiro, Brazil

## Abstract

In recent decades temperature elevation has been the focus of many studies on climate change, including effects on planktonic communities, but few studies have examined the effects of increased water color ("brownification"). Since these changes are likely to occur simultaneously, it is important to investigate their potential interactive effects. Accordingly, we performed a mesocosm experiment where we combined a 3°C increase in temperature with a doubling in water color to study how these factors affect zooplankton. In particular, we looked at recruitment of cladocerans and copepods from the sediment in spring, as well as their establishment in the water column. Our results show that an elevated temperature will have considerable effects on recruitment as well as on pelagic abundances of both cladocerans and copepods, whereas increases in water color will have less effects on the recruitment and pelagic establishment. But more importantly, the proportion of cladocerans in the water column, relative to copepods, increased at higher temperature, suggesting that cladocerans benefit more from elevated temperatures than copepods do. Overall, these results likely stem from the combined effect of changes in recruitment and differences in life history between copepods and cladocerans. Taking a wider outlook, this suggests that future climate warming will change the dominance pattern of zooplankton communities in spring, and, in accordance with the experimental data, we here show that cladocerans are more abundant than copepods in natural lake ecosystems during warmer rather than cooler years.

## Introduction

During the past 100 years the global mean air temperature has risen at an accelerating rate and is expected to continue to increase, gaining between 2 and 5°C in northern Europe over the next century [1], [2]. This rise is not uniform, however; more pronounced increases are expected to occur in winter and spring [1], and spring is a time critical for the shaping of zooplankton communities.

Concurrently with elevated temperatures, many lakes have been subject to additional changes such as increased dissolved organic carbon (DOC) and its associated increase in water color [3], [4]. In many northern temperate lakes this “brownification” [5] has resulted in a doubling in water color over the past 20–30 years, and this trend is likely to continue (Hansson, unpublished). Although separate studies have focused on elevated temperature [6]–[8] or increased water color [9], few studies have addressed the potential interactive effects these stressors might have on planktonic communities, but see [10], [11].

In temperate lakes the spring development among many zooplankton taxa generally starts with the hatching of individuals from resting eggs and from emerging dormant instars [12], [13]. Timing of the hatching of these eggs and the breaking of dormancy has been shown to depend mainly on abiotic factors, and important among them are water temperature and light availability [14]–[17]. Once recruited, cladocerans and copepods have different life-history strategies. Most cladocerans are parthenogenetic and thereby are able to rapidly reproduce during periods of favorable conditions. On the other hand, copepods reproduce only sexually and need to go through a series of post-embryonic developmental stages before they reach adulthood and are able to reproduce [18]. The rate of reproduction (as well as recruitment) is also related to water temperature, with higher growth and development rates at higher temperatures. Some previous studies investigating temperature effects on zooplankton have shown that increased temperatures lead to earlier peak abundances of some zooplankton [7], [11], but other studies have demonstrated this correlation to be lacking for some zooplankton [19]. Copepods and cladocerans both recruit from resting stages at the sediment surface [12], [14], [20], and it is of considerable interest to investigate how future climate change affects these factors that influence recruitment. Such knowledge would improve the capacity to predict future climate-change-induced trends in the dominance relationships of zooplankton and changes in patterns of zooplankton community development.

Since temperature escalations due to climate change are likely to co-occur with increases in water color, we sought to address potential interactive effects. To this end, we performed a mesocosm experiment where both temperature and water color were manipulated, simulating the projected future scenario for temperate lakes. We hypothesized that an elevated temperature would lead to an earlier recruitment of all zooplankton groups and also an earlier establishment of the pelagic population. Water color was hypothesized to have a negative effect on zooplankton recruitment since it would reduce the light stimulus for hatching. Furthermore, we were interested in determining if water color would interact with temperature in such a way that the set points of the cues for recruitment, with respect to light availability and temperature, would change compared to those under present conditions. If so, they could change the pattern of zooplankton recruitment and thereby also the establishment of them in the water column.

## Methods

### Sediment Collection

Sediment was collected from Lake Krankesjön (N55° 42′ 27′′, E13° 27′ 58′′), a lake in southern Sweden, in the beginning of February 2010. The top few centimeters of sediment were collected with hand nets through holes in the ice from approximately 1 m depth; this pooled material was transported to the laboratory where it was stored in a dark cold-room at 2.8°C, a temperature corresponding to the water temperature at the outlet of the lake at the time of sampling.

### Experimental Set-up

An outdoor mesocosm experiment, consisting of 24 insulated polyethylene enclosures (diameter = 0.7 m, height = 1 m), was run between March 23 and May 24, 2010. The four treatments consisted of (C) controls mimicking the concurrent state in Krankesjön with respect to temperature and water color (see Hansson et al., 2007, for more information about Krankesjön [21]); (T) an increase in temperature of 3°C compared to the control; (B) a doubling in water color compared to the water color of Lake Krankesjön, measured as light absorbance at 420 nm; and (TB) a combination of both factors, which constituted a future scenario with respect to temperature and humic content. Each of the four treatments was replicated six times.

All experimental enclosures received 60 mm of mixed lake sediment as well as 400 L of lake water. In the treatments with increased water color, 20% of the initial volume of water was replaced with lake water from the humic Lake Liasjön, a lake situated in southern Sweden (N56° 26′ 47′′, E13° 59′ 34′′). Extrapolating monitoring data, this 20% addition of humic water to the Lake Krankesjön water, mimicked the projected change in water color that can be expected in temperate lakes within 25–75 years (Hansson et al., unpublished data). All water added to the enclosures was filtered through a 20- µm mesh to eliminate zooplankton, i.e. to make sure that all zooplankton originated from the sediment. After adding the brown water, a sample for pelagic zooplankton abundance was taken from each enclosure. No cladocerans nor copepodids and adult copepods were present in the water at this sampling, and there were no significant differences in nauplii abundance (ANOVA: F_3, 20_ = 1.41 p = 0.27), thereby confirming that the filtration process was efficient in removing zooplankton from both the clear and the brown water. Treatments with increased temperature were heated with aquarium heaters (Jäger, 150 W). These were controlled by a computer system that measured the temperature in the controls and in the temperature-elevated treatments once every 10 seconds using temperature sensors (National Semiconductor, LM335AZ, precision temperature sensor). The control system then regulated each temperature-elevated enclosure individually based on the mean of the controls at each measurement time (see Nicolle et al., 2012, for more information about the temperature system [11]). The ambient-temperature enclosures (C) went from 5.9°C, at the first sampling occasion, to 17.2°C, at the last sampling, with a peak temperature of 19.8°C at the end of May. The temperature-elevated enclosures (T & TB) followed the same temperature pattern with a mean temperature difference (± SD) of 3.0±0.02°C compared to the ambient-temperature enclosures. For more information regarding the physical and chemical conditions of the treatments see Table 1. All enclosures were gently aerated using aquarium pumps to avoid temperature stratification. Evaporated water from the enclosures was replaced weekly with distilled water to maintain a constant volume and conductivity. Moreover, to maintain a constant level of water color in the B and TB treatments, weekly additions of 20-µm-filtered humic water were made throughout the experiment. The walls of the enclosures were gently scrubbed once a week to remove periphyton.

**Table 1:** Physical & chemical data.

	C	T	B	TB
Absorbance	0.0088±0.0003	0.0087±0.0003	0.0198±0.0005	0.0197±0.0005
pH	8.2±0.03	8.2±0.02	8.3±0.03	8.2±0.02
Totalphosphorous	18.4±3.4	16.2±2.1	16.3±0.9	16.8±4.2
Chlorophyll-*a*	6.0±0.7	6.1±0.4	6.2±0.5	6.9±0.6

Mean (±SD) absorbance (λ420 nm), pH, total phosphorous (µg/L), and chlorophyll-*a* content (µg/L) in the enclosures during the experimental period (22 March - 27 May, 2010).

### Sampling and Analysis

Enclosures were sampled once a week using a Plexiglas tube (length = 1 m, diameter = 70 mm) to get an integrated water sample. Three tube samples were taken along the diameter of each enclosure, and from this pooled sample water was taken for absorbance analysis, chlorophyll-*a*, pH, nutrients and zooplankton abundance. Absorbance at 420 nm was measured with a spectrophotometer (Beckman Coulter, DU800) on GF/C-filtered water to monitor water color changes. Chlorophyll-*a* was analyzed on a flourometer after extraction in 95% ethanol (TD 700, Turner Design). Zooplankton were sampled using a 50- µm plankton mesh and were preserved with Lugol's acid solution for later enumeration at 20× magnification (Olympus SZ-40).

After each sampling of the water column, i.e. once a week, traps (0.008 m^2^) for retrieving zooplankton were set at the sediment surface to sample the zooplankton recruitment from the sediment to the water. The traps consisted of a jar and a funnel attached to a plastic frame with two 10- µm net windows to allow circulation and exchange of water between the trap and the surroundings [22]. Prior to setting, the trap was filled with tap water in order to avoid contamination by plankton from the water column. When lowered, a 10-µm net was held in front of the opening to minimize the risk of any plankton entering the trap during deployment. This ensured that everything larger than 10 µm present in the trap at the end of the sampling period originated from the sediment surface. Traps were retrieved after 48 hours and the samples preserved with Lugol's acid solution for later enumeration. Recruited macro-zooplankton were counted in the entire sample volume (60 mL) to get a more accurate measurement of the recruitment. Since we focus here on recruitment from the sediment to the water column, strictly benthic cladoceran species were removed from further analysis; these included Chydorids and *Eurycercus*, as well as occasional adult and egg-bearing individuals of other cladoceran species that could not have originated from resting stages. Hence, our analysis includes only true pelagic species–copepods (focusing on cyclopoid copepods as they constituted more than 99% of all copepods in the study), *Bosmina*, and *Ceriodaphnia*–of which the latter two were pooled and categorized as cladocerans. Samples from the water column were handled and categorized in the same way, although the analysis also included the adult and egg-bearing cladocerans.

### Field Data

Using monitoring data (pelagic samples) for more than ten years from Lake Krankesjön and Lake Ringsjön, we calculated the cladoceran to copepod ratio as a way to assess if between-year fluctuations in spring temperature had any effect on the dominance pattern between these taxa during May, which we observed in our experiment. We used the mean air temperature two weeks prior to each sampling occasion as a standardized proxy for lake temperature that the zooplankton would have experienced prior to sampling and that could have been expected to affected pelagic reproduction around the sampling occasion. No specific ethical permits were required for the described studies. According to Swedish law, no specific permits were needed for the field studies.

### Statistics

The effects of temperature and water color on zooplankton recruitment and pelagic abundance were evaluated using repeated-measures ANOVAs; for this we used log_10_-transformed data to satisfy the assumptions for parametric tests. Furthermore, we applied the Greenhouse-Geisser correction to the statistics in order to account for any lack of sphericity. To examine the relative proportions of copepods versus cladoceran zooplankton, we calculated, using all samples, the ratios for mean recruitment and for pelagic abundance during the experimental period. These ratios were analyzed with a two-way MANOVA–with temperature and color as fixed factors; and ratio, for both recruitment and pelagic abundance, as dependent variables. The ratios were log_10_ transformed before analysis to fulfill the assumptions for parametric tests. To assess the magnitude of the treatment effects, we calculated the effect size for the mean pelagic abundances and for the cumulative recruitment, pooling the entire sampling period. These effect sizes are from here on referred to as “season-integrated effect size” because the values represent the response given during the entire experimental period (season). By using effect size, the responses of the treatments are standardized in relation to the control. It incorporates the mean value and the standard deviation of both the control and the treatment as well as the number of replicates, thereby condensing the results of the different replicates to one response value for the treatment in relation to the control [23].

Ratios from the field data were log_10_-transformed to fulfill the assumptions for parametric test. Data were analyzed using independent-sample t-test using the overall mean temperature for all years included (11.48°C) as a cutting point for group categorization i.e. putting a year as being either above or below the overall mean temperature. Statistical analyses were made using Statistical Package for the Social Science (SPSS) 19 for Macintosh.

## Results

### Copepods

**Figure 1:**
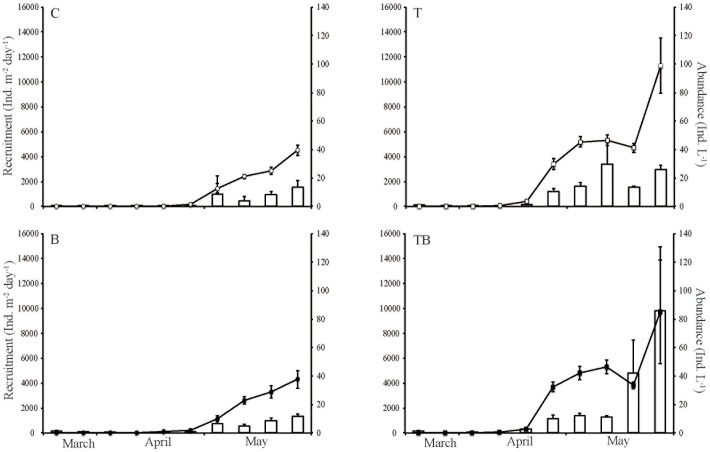
Copepod recruitment and abundance. Mean (+SE) recruitment rates (bars) and abundance in the water column (lines) of copepods during spring in controls (C), temperature-elevated (T), increased water color (B), and the combined treatment (TB) illustrating a future scenario. The increase in mean pelagic abundance in the temperature-elevated treatments on the last date represents the occurrence of a new generation as small copepodids dominated this sampling occasion.

Both copepod recruitment and abundance in the water column increased with time in all treatments and also increased earlier in those treatments with elevated temperatures (T and TB) (Table 2). An increased temperature also had a positive effect on the average number of individuals both recruited and present in the water column (Table 2, Fig. 1). A threshold for abundance in the water column (the point when the pelagic population mean for that treatment reached more than one individual per liter) occurred one week earlier in the elevated-temperature treatments compared to the ambient-temperature treatments (Fig. 1). The addition of brown water did not significantly affect the recruitment and pelagic establishment over time nor the average levels of individuals in the treatments (Table 2). Furthermore, there was no interaction between temperature and water color in any of the measured variables (Table 2). This pattern was also mirrored in the season-integrated effect size of both the copepod recruitment and the copepod pelagic abundance–i.e. a higher season-integrated effect size in treatments with elevated temperature, and a lower season-integrated effect size in the brown-water treatment (Table 3).

**Table 2:** Repeated-measures ANOVAs.

	Copepods				Cladocerans			
Factor	Recruitment		Abundance in		Recruitment		Abundance in	
			Water Column				Water Column	
a) Within-Subjects Effects	F_4.75, 95.0_	p	F_2.96, 59.1_	p	F_4.82, 96.5_	p	F_3.39, 67.8_	p
Time	94.5	**<0.001**	591	**<0.001**	17.2	**<0.001**	495	**<0.001**
Time × Temp.	7.9	**<0.001**	36.2	**<0.001**	5.38	**<0,001**	83.9	**<0.001**
Time × Color	0.81	0.54	0.73	0.54	0.37	0.86	2.66	**0.05**
Time × Temp. × Color	1.55	0.18	0.52	0.70	0.97	0.44	1.88	0.13
b) Between-Subjects Effects	F_1, 20_	p	F_1, 20_	p	F_1, 20_	p	F_1, 20_	p
Temp.	22.8	**<0.001**	208	**<0.001**	25.2	**<0.001**	489	**<0.001**
Color	0.91	0.35	0.23	0.64	3.59	0.07	4.00	0.06
Temp. × Color	0.37	0.55	1.82	0.19	3.77	0.07	0.50	0.49

Summary of the statistics from the repeated-measures ANOVAs. Section a) shows Greenhouse-Geisser corrected statistics over the within-subjects effects and section b) shows the between-subjects effects on the average number of individuals during the experiment. Values in bold indicates significant differences at the p<0.05 level.

### Cladocerans

**Figure 2:**
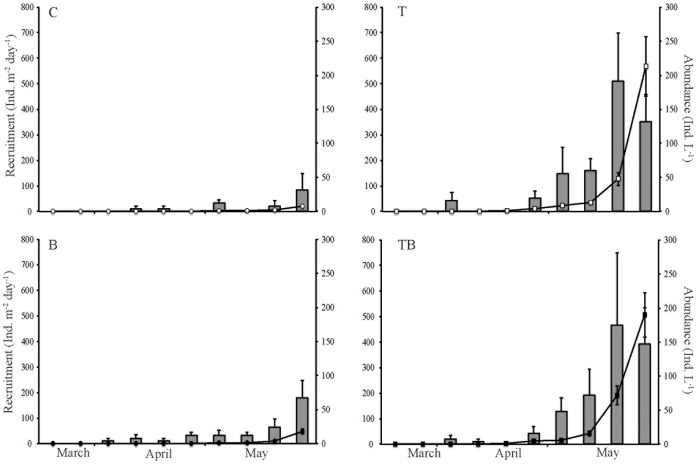
Cladoceran recruitment and abundance. Mean (+SE) recruitment rates (bars) and abundance in the water column (lines) of cladocerans during spring in controls (C), temperature-elevated (T), increased water color (B), and the combined treatment (TB) illustrating a future scenario.

The recruitment and pelagic abundance of cladocerans followed the same pattern as the copepods, with an earlier increase for treatments with elevated temperatures (Table 1, Fig. 2). Cladoceran presence in the water column, estimated in the same way as for the copepods, occurred one week earlier in the elevated-temperature treatment (T) and two weeks earlier in the future-scenario treatment (TB) (Fig. 2). Furthermore, an elevated temperature also had an augmenting effect on the average number of individuals both recruited from the benthic habitat and present in the water column (Table 2). Water color had no effect on the recruitment patterns over time nor on the average number of individuals recruited (Table 2). The increase in water color showed a moderate effect on the pelagic establishment of cladocerans over time (Table 2) and also moderately affected the average level of individuals in the water column (Table 2). There were no interactions between water color and temperature for recruitment or for pelagic abundance (Table 2). In contrast to the RM-ANOVA, which did not show any effects of water color on cladoceran recruitment, there was a slight positive season-integrated effect size on the cumulative recruitment (Table 3). Also, an elevated temperature led to a larger season-integrated effect size on recruitment in both the T and the TB treatments. The season-integrated effect size for the pelagic abundance of cladocera followed the same pattern as the recruitment except that the TB treatment showed higher values compared to the T treatment (Table 3).

**Table 3:** Season-integrated effect size.

	Copepods		Cladocerans	
Treatment	Recruitment	Abundance in WaterColumn	Recruitment	Abundance in Water Column
Temperature (T)	1.9	4.4	2.5	4.0
Brown (B)	0.0	0.1	1.2	1.8
Temperature and Brown (TB)	1.5	2.9	2.4	6.5

Season-integrated effect size for recruitment and abundance in the water column for both copepods and cladocerans in the different treatments. An effect size of zero means that there were no effect of the treatment in relation to the control whilst the higher the value the bigger the effect.

### Copepods vs. Cladocerans

**Figure 3:**
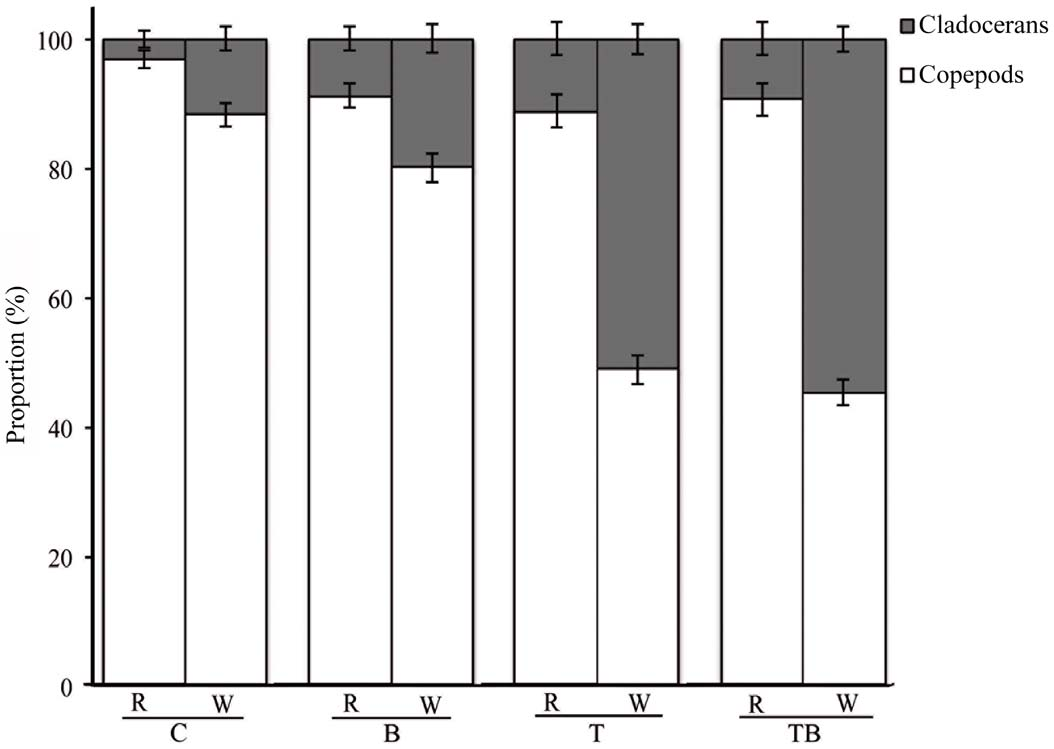
Copepods vs. cladocerans in the experiment. Mean proportions (±SE) of copepods (white) and cladocerans (gray) for the mean recruitment (R) and abundance in the water column (W) in each treatment during the experimental period.

There was an overall effect of elevated temperature on the ratios between cladocerans and copepods (MANOVA: Wilks’ Lambda = 0.076, F_2,19_ = 114.98, p<0.001). Looking at the separate effects, the increase (due to temperature) in the clodeceran-to-copepod ratio for recruitment was smaller (F_1,20_ = 4.16, p = 0.055), but a larger increase due to temperature was found for pelagic abundance in both temperature-elevated treatments (T & TB) (F_1,20_ = 241.59, p<0.001). In the water column, the proportions of cladocerans to copepods in the these treatments (T and TB) constituted an almost 50∶50 ratio, while they were near 10∶90 in the controls and 20∶80 in the brown ambient-temperature treatment (on a season-long basis) (Fig. 3). The relative proportions in both recruitment and abundance were not affected by water color alone (MANOVA: Wilks’ Lambda = 0.787, F_2, 19_ = 2.57, p>0.05) nor were there any interactive effects between temperature and water color (MANOVA: Wilks’ Lambda = 0.870, F_2, 19_ = 1.415, p>0.05). Hence, the MANOVA shows that there is an effect on the proportion for the pelagic population in both the T and TB treatments, but that this difference is driven by the change in temperature alone and not by the combination of elevated temperature and water color in the TB-treatment, as both the T and TB respond in the same way regardless of color in relation to the control treatment.

### Field Data

**Figure 4:**
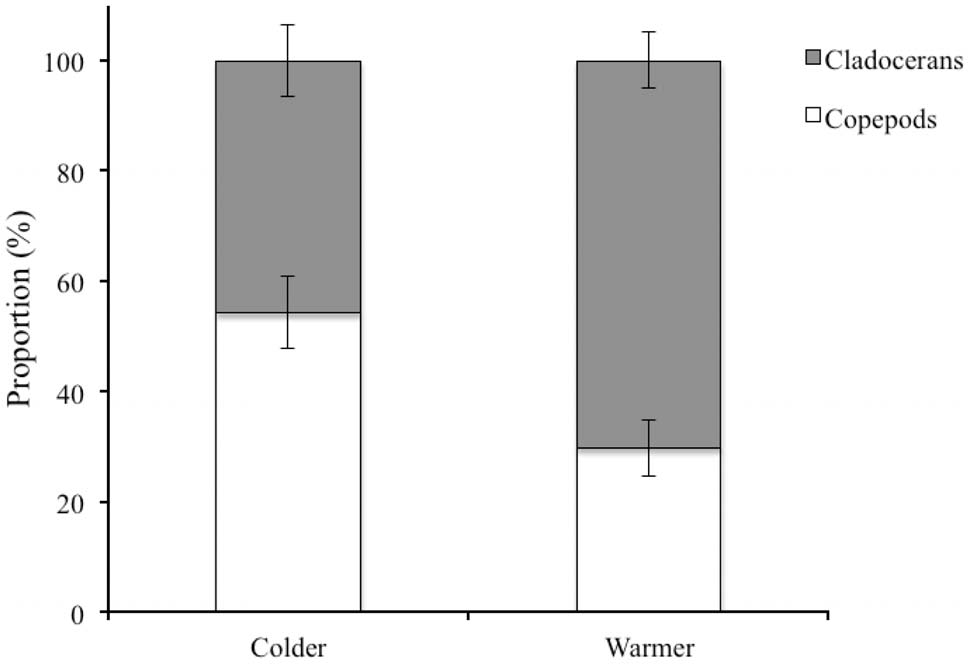
Copepods vs. cladocerans in the field. Mean (±SE) proportion of copepods (white) and cladocerans (gray) observed in over ten years of field data from Lake Krankesjön and Lake Ringsjön during springs colder and warmer than the overall mean temperature in May (11.48°C) of all years investigated.

There was a significantly higher cladoceran versus copepod ratio in the lakes investigated during years that had a higher temperature than the overall mean, i.e. years with a mean temperature in May higher than 11.48°C (independent-sample t-test: t_20_ = 3.24, p<0.01) (Fig. 4).

## Discussion

Many studies have investigated effects of climate change on pelagic zooplankton populations. This study is the first, however, to demonstrate that climate change affects not only the pelagic population but also the recruitment from the benthic habitat. There have been, however, previous studies proposing that similar pelagic effects might involve recruitment as a potential driver [6]. The recruitment (emergence from the benthic habitat) of copepods and cladocerans increased at elevated temperatures; but the relative proportions in recruitment of the two taxa were only marginally affected, with a slightly higher proportion of cladocerans at elevated temperatures. Even so, this last-mentioned effect appears to be of crucial importance. In the water column this marginal increase in the proportion of cladocerans recruited (likely in combination with higher reproductive rate) led to a more substantial increase in proportion of cladocerans in the pelagic population. This suggests that differences in life history strategies, with a shorter reproductive cycle, will favor cladocerans compared to copepods in a future climate-change scenario. At elevated temperatures the small proportion of recruited cladocerans had the ability to overtake the large proportion of recruited copepods and reach similar proportions in the water column. We argue that differences in reproductive strategy (parthenogenesis vs. sexual reproduction) are likely the main mechanism behind the increase in proportion of cladocerans at elevated temperatures, which has also been noted in previous studies [7]. This was also seen in our field data where we observed a higher cladoceran versus copepod ratio during warmer years compared to colder ones. Since we do not have any data for recruitment from the field we cannot disentangle whether these differences are due to differences in recruitment, reproduction or both. Furthermore, Adrian, et al. (2006) noted that fast-growing zooplankton (*Daphnia* and *Bosmina*) displayed a synchronous response to elevated temperatures in spring and that they advanced their peak abundances by several weeks during periods with higher temperature. These findings are in agreement with the results of this study, where an elevated temperature advanced the occurrence (although not peak abundance) in the water column by 1–2 weeks. However, in addition to a higher reproductive rate, we here add the mechanism that an earlier establishment in the water column is also a result of earlier and more pronounced recruitment rates from the sediment, thereby speeding up the rate of succession towards cladoceran dominated communities. Our findings that cladocerans benefit from elevated temperatures are contrary to what Winder and Schindler (2004) found for *Daphnia* in Lake Washington. They observed a decrease in the *Daphnia* population during a period of warming and argued that this might have been because the hatching of *Daphnia* was unaffected by the increased temperature [19]. In contrast, we observed an increase in cladoceran recruitment in the experiment at elevated temperatures and with that an increased dominance in pelagic abundance. A possible explanation to the differences may be that different species respond differently to elevated temperatures. In accordance, it has been shown that (both on a global and a micro-geographical scale) even different clones from the same species can react differently to cues for hatching [17], [24], [25]. This suggests a need for more studies investigating different zooplankton communities, different latitudes, and encompassing inter- and intra-specific variations on a global scale. Through these studies we can get a more accurate picture of what we can expect to find in lakes in the future.

As expected, elevated temperatures had an amplifying effect on both zooplankton recruitment and pelagic abundance. Looking at water color, when only this variable was increased (B treatment), cladoceran recruitment and pelagic abundance rose, but copepods remained unaffected. For cladoceran recruitment we found a positive season-integrated effect size for the B treatment (increased color). However, we expected the opposite, reasoning that increased color would reduce the intensity of light at the sediment surface, thereby delaying the hatching of cladocerans. Conversely, the results were consistent with what we expected for copepods, which were unaffected by increased water color. This latter result is not surprising since light is more often connected with the induction of diapause in copepods, rather than with its termination, although there are exceptions [14], [15]. Temperature often functions as a terminating cue for diapause in copepods [26] and cladocerans, supporting the high season-integrated effect sizes found for both groups in the temperature-elevated treatments. While the season-integrated effect size on cladoceran recruitment was positive in both the B and T treatments, there were no additive effects. Rather, the TB treatment responded similarly to the T treatment, with similar season-integrated effect size levels, indicating that the effect of water color is weak. Moreover, our data suggest that the overall effect of increased water color is weaker than for projected temperature changes. Extending from these results to a climate-change point of view, projected water-color changes might be expected to cause a weaker effect compared to temperature.

Cladocerans in the water column were most affected by increased temperature and especially by the combination of the two factors as shown by the high season-integrated effect size in these treatments. The higher effect size in the TB treatment compared to the T treatment was not a result of higher season-integrated abundances of cladocerans in this treatment (T = 28.8 and TB = 28.9 individuals per liter), but rather a result of a lower variance in the TB treatment compared to the T treatment (see ± SE in Fig. 2). In other words, there was a more consistent response among the cladocerans in the TB treatment. It has been shown that the allochthonous carbon in humic water is able to support zooplankton [27], [28]. If this was the case in our enclosures it could explain the more consistent pattern (lower variance) in the temperature-elevated brown treatment (TB). The zooplankton in this treatment were then not relying only on the phytoplankton as food, but could also utilize material from the microbial loop (i.e. bacteria) during times of low algal food availability. This could also have been the case in the brown water treatment (B), but since the difference in effect size between cladoceran recruitment and abundance was rather small in this treatment the increased abundance was likely mainly a result of the increased recruitment rather than increased reproduction rate. However, this analysis does not apply to the copepods, which showed the highest season-integrated effect size, with respect to abundance, in the temperature-elevated treatment and not in the TB treatment.

One should be careful when scaling up results from experiments to natural ecosystems, since there are many different factors that are at work in nature that are not mimicked in experimental set-ups [29], [30]. However, experiments allow us to study specific parts of the spring phenology in lakes and, as shown in this experiment, allow us to pinpoint the potential mechanisms behind observed responses, such as earlier emergence and differences in reproductive strategies, so that we can make quantitative predictions of how our future lakes may function.

In conclusion, our data clearly show that climate change, and especially changing temperature, is likely to affect zooplankton emergence and also their establishment in the water column in spring. Furthermore, zooplankton taxa that are able to reproduce rapidly via parthenogenesis respond more rapidly to an elevated temperature compared to taxa with more complex life-history strategies, which ultimately could affect the competition between these taxa in spring. This is also shown in the field data, which clearly show that years with higher temperature do in fact favor cladocerans in spring. A potential consequence of stronger recruitment and faster reproductive rate, and the fact that cladocerans generally are considered to be the more efficient grazers [31], [32], is that the timing and duration of the spring clear-water phase may be predicted to change in the future of these freshwater ecosystems [33].
